# High place phenomenon, obsessive-compulsive symptoms and suicidality

**DOI:** 10.3389/fpsyt.2025.1653961

**Published:** 2025-10-30

**Authors:** Zahra Asgari, Azam Naghavi, Ali Abbasi, Andrea Ertle, Lara Wiesmann, Tobias Teismann

**Affiliations:** ^1^ Department of Counseling, Faculty of Education and Psychology, University of Isfahan, Isfahan, Iran; ^2^ Department of Political Sciences, University of Isfahan, Isfahan, Iran; ^3^ Institute of Psychology, Humboldt-Universität zu Berlin, Berlin, Germany; ^4^ Mental Health Research and Treatment Center, Ruhr-Universität Bochum, Bochum, Germany; ^5^ DZPG (German Center for Mental Health), Partner Site Bochum/Marburg, Bochum, Germany

**Keywords:** high place phenomenon, suicidal ideation, obsessive-compulsive symptoms, anxiety sensitivity, call of the void

## Abstract

**Background:**

The high place phenomenon (HPP), referring to a sudden urge to jump when standing in a high place, occurs frequently in both suicidal and non-suicidal individuals. Despite apparent similarities, researchers have not yet examined potential associations with obsessive-compulsive symptoms, nor has the phenomenon been explored in a non-Western society.

**Methods:**

The study comprises two samples of Iranian adults: An online sample including *N* = 257 participants (54% male; *M*_age_=37.03, *SD*_age_= 11.51) and a sample of mobility impaired participants including *N* = 233 participants (56.2% male; *M*_age_=37.84, *SD*_age_= 9.75, range: 18–68 years). All participants filled out questionnaires on experiences with the high place phenomenon, depression, suicidal ideation/behavior, anxiety sensitivity and obsessive-compulsive symptoms.

**Results:**

Between 39% and 62% of participants reported being familiar with the HPP. In both samples, obsessive-compulsive symptoms showed a significant association with the severity of the phenomenon, even after accounting for depression, suicidal ideation/behavior, and anxiety sensitivity. The presence of the HPP was only associated with obsessive-compulsive symptoms in one of the two samples.

**Conclusion:**

Findings point to the cross-cultural nature of the HPP. Furthermore, the association between obsessive-compulsive symptoms and the HPP speaks to a conceptualization of the HPP as being part of the phenomenological field of (subsyndromal) OCD symptoms.

## Introduction

The high place phenomenon (HPP) describes the experience of a sudden impulse or urge to jump when in a high place: “Whenever I’m standing on a bridge or near a balcony, I suddenly think, ‘What if I jump?’ It’s so scary, and I don’t want to do it, but I can’t shake the thought”. Studies conducted in Germany (1, 2) and the USA (3) have demonstrated the prevalence of the phenomenon, with 43% to 60% of participants affirming lifetime experiences with the phenomenon. Interestingly, the phenomenon is known to participants with lifetime suicidal ideation as well as participants who deny having ever suffered from suicidal ideation in their life (1–3): Therefore, the HPP is not necessarily an indicator of a suicidal crisis. Hames et al. (3) rather postulate that the HPP can be understood as a misinterpretation of a safety or survival signal (“Back up, you might fall”) that is intended to keep a person alive and out of danger. Positive associations between the HPP and anxiety sensitivity – that is, the tendency to be fearful of anxiety-related symptoms and arousal sensations (4) – offer some support for this idea. Accordingly, Hames et al. (3), assume that “individuals who tend to be more sensitive to such safety signals will be more likely to report experiencing the phenomenon, as compared to individuals who are not as sensitive to safety signals” (p. 1115). However, not all studies on the HPP have found a positive association between the HPP and anxiety sensitivity (1, 2). Therefore, it is possible that the HPP does not fulfill a life-preserving purpose but rather represents a mundane type of an unwanted intrusive thought.

Unwanted intrusive thoughts are defined as unintended thoughts, images, ideas or impulse which are associated with negative affect and difficult to control (5). An example of unwanted intrusive thoughts are obsessions. Obsessions primarily concern violence (“What if I accidentally harm someone while driving?”), sex (“What if I am secretly attracted to someone of the same sex, even though I don’t feel that way?”), religion (“What if I’m not praying correctly and God will be angry with me?”), mistakes (“I’m terrified I’ll make a mistake and it will ruin my future.”), and the possibility of being responsible for causing (or not preventing) harm (6). A specific subtype of obsessions are suicidal obsessions, which involve recurrent, unwanted, intrusive and distressing thoughts focused on one’s suicide (e.g., intentionally walking in front of a bus while crossing the street). Suicidal obsessions are typically experienced as alien to one’s sense of self or values (i.e., ego-dystonic) and elicit strong, distressing feelings, such as anxiety, fear, shock, and self-doubt (7). In general, unwanted intrusive thoughts are often triggered by stimuli in the environment (such as knives or being in a high place) and sometimes these unwanted intrusive thoughts are accompanied by distressing physical responses, that is, experiences that seem like urges to act on these violent intrusions (6).

Unwanted intrusive thoughts lie at the heart of the cognitive-behavioral conceptualization of obsessive-compulsive disorders (OCD; 8). However, unwanted intrusive thoughts are not exclusive to individuals with OCD. Studies (8–10) have demonstrated that such thoughts are common in the general population: In these studies 80-93% of non-clinical participants affirmed experiences with unpleasant and unwanted intrusive thoughts and impulses (e.g., “Impulse to blurt out curse words when everyone is praying silently in the synagogue”). Unwanted intrusive thoughts, including those with sexual, aggressive, or blasphemous content, are therefore a normal part of life. The same might apply to the HPP. To date, no research has examined the link between HPP and obsessive-compulsive symptoms. Consequently, it remains unclear whether HPP should be considered part of the phenomenological spectrum of (subsyndromal) OCD symptoms.

Against this background, the first aim of the present study was to investigate the association between OCD symptoms and the HPP. A second aim of the current study was to examine the phenomenon in a different cultural context as all three studies on the phenomenon so far have been conducted in Western societies (1–3). Therefore, it is unclear to what extent findings can be generalized to Asian societies, or more specifically to the Iranian society, which exhibits considerable cultural differences to German and American society (11), for example regarding individualism/collectivism, communication style, gender roles, family structure and the role of religion.

In the present research project, we had the opportunity to study the phenomenon in two independent *ad hoc* samples of Iranian adults: an online sample aimed at the general population and a sample of individuals with a mobility impairment. There were no differential expectations associated with the two samples; rather, the study aimed to determine whether HPP is a phenomenon that can be observed in different social groups. We expected that the HPP is prevalent in both samples and that the severity and the presence of the HPP is associated with OCD symptoms in both samples, even after controlling for age, gender, suicidal ideation/behavior, depression and anxiety sensitivity. Previous studies have shown that all of these control variables, except for gender, are associated with the HPP (1–3). Gender was included because OCD symptom expression seems to differ between males and females (12).

## Methods

### Participants and procedure

#### Sample 1 (online sample)

The sample comprised *N* = 257 participants (54.0% male; *M*_age_=37.03, *SD*_age_= 11.51, range: 18–80 years) who took part in an online assessment between July and October 2023. One-hundred thirty-nine participants (54.1%) reported lifetime suicidal ideation/behavior (SBQ-Item 1 score ≥2), 73 participants (28.4%) reported some suicidal ideation within the last 12 months (SBQ-R Item 2 score ≥1) and 16 participants (6.2%) indicated that they had attempted suicide at least once in their lifetime. Most participants were either married (52.1%, *n* = 134) or single (42.0%, *n* = 108) and most participants had either a Bachelors’ degree (38.9%, *n* = 100) or a Masters’ degree (30.7%, *n* = 79).

#### Sample 2 (mobility impaired sample)

The sample comprised *N* = 233 participants (56.2% male; *M*_age_=37.84, *SD*_age_= 9.75, range: 18–68 years) who took part in an online assessment between January and March 2024. One-hundred thirty-four participants (57.5%) suffer from an acquired mobility impairment and *n* = 99 (42.5%) from a congenital mobility impairment. One-hundred and four participants (44.6%) reported lifetime suicidal ideation/behavior (SBQ-Item 1 score ≥2), 73 participants (31.3%) reported some suicidal ideation within the last 12 months (SBQ-R Item 2 score ≥1) and 19 participants (8.2%) indicated that they had attempted suicide at least once in their lifetime. Most participants were either single (62.2%, *n* = 145) or married (34.3%, *n* = 80) and most participants had either a Bachelors’ degree (27.0%, *n* = 63) or a Masters’ degree (42.9%, *n* = 100).

Data in both studies were collected through an anonymous online survey using the Porsline-server (https://porsline.ir). Participants were recruited through postings at local universities (Sample 1) or postings at institutions for individuals with a mobility impairment (Sample 2). To participate in the study, individuals had to be at least 18 years old and provide informed consent at the outset. Before completing the assessments, all participants received information about the study’s aims, their voluntary participation, and details concerning data storage and confidentiality. The Ethics Committee at the University of Isfahan in Iran reviewed and approved the study (IR.UI.REC.1402.061 and IR.UI.REC.1399.008).

### Measures

#### High place phenomenon index (HPPI)

The HPPI (3) assesses with three items how frequently participants have experienced the high place phenomenon in their lifetime (e.g., “When standing on the edge of a tall building or walking on a bridge, have you ever had the urge to jump?”), using a 5-point Likert type scale ranging from (0) never to (4) always. The original scale evidenced good internal consistency: *α* = .85 (3). The Farsi version of the HPPI was developed using a translation-back-translation procedure according to relevant guidelines for the translation of psychometric instruments (13). Internal consistencies of the Farsi HPPI were good in the Online Sample (*α* = .85) and the Mobility Impaired Sample (*α* = .84).

#### Patient health questionnaire (PHQ-9; Farsi version)

The Patient Health Questionnaire (PHQ-9) (14, 15) assesses symptoms of depression (e.g., lack of interest, feeling down, poor appetite) with nine items (e.g., “Over the last two weeks, how often have you been bothered by any of the following problems: Little interest or pleasure in doing things?”) that are rated on a 4-point Likert-type scale (0=not at all, 3=nearly every day). Internal consistency of the PHQ-9 has been shown to be high [*α* = .86 to.89; (14)]. Internal consistency was good (*α* = .88) in the Online sample, as well as in the Mobility Impaired Sample (*α* = .89).

#### Suicide behaviors questionnaire-revised (SBQ-R; Farsi version)

The SBQ-R (16, 17) comprises four items assessing different facets of suicidal ideation and behavior. In the current study items 1 and 2 of the SBQ-R were used. Item 1 of the SBQ-R measures lifetime suicidal ideation, suicide plans and attempts. The item (“Have you ever thought about or attempted to kill yourself?”) is rated on a 6-point Likert-type scale (1=never, to 6=I have attempted to kill myself, and really hoped to die). Item 2 of the SBQ-R assess frequency of suicidal ideation within the last 12 months (“How often have you thought about killing yourself in the past year?”). The item is rated on a 5-point Likert scale from 1 (never) to 5 (often). The SBQ-R has been recommended for screening purposes and has repeatedly been used in clinical and non-clinical samples (16).

#### Anxiety sensitivity index (ASI; Farsi version)

The ASI (18, 19) is a 16-item self-report inventory designed to measure the degree to which individuals are concerned about the potential negative effects of experiencing anxiety symptoms (“It scares me when I feel faint”, “Unusual body sensations scare me”). Respondents are asked to indicate the degree to which each item applies to them using a 5-point Likert type scale ranging from (0) very little to (4) very much. The scale has been found to have strong internal consistency and test-retest reliability [e.g., (20)]. Internal consistency was good within the Online sample (*α* = .88) and the Mobility Impaired Sample (*α* = .93).

#### Obsessive-compulsive inventory – revised (OCI; Farsi version)

The OCI-R (21–23) is an 18-item tool which assesses six clusters of obsessive-compulsive symptoms including washing, checking/doubting, obsessing, mental neutralizing, ordering, and hoarding (e.g., “I find it difficult to control my own thoughts”; “I frequently get nasty thoughts and have difficulty in getting rid of them”). Each item of the OCI-R is rated on a 5-point Likert scale from 0 (not at all) to 4 (extremely). The Farsi version of the questionnaire was shown to have good internal consistency [*α* = .95; (23)]. Accordingly, internal consistency was good in the Online sample (*α* = .88 and the Mobility Impaired Sample (*α* = .88).

### Statistical analyses

Statistical analyses were conducted with SPSS 29. Descriptive statistics and zero-order bivariate correlations between the investigated variables were calculated. Furthermore, group differences between lifetime suicidal ideators (SBQ-R, Item 1-score ≥ 1) and non-ideators (SBQ-R, Item 1-score = 0) were investigated using t-tests. To identify significant predictors of the severity of experiences with the high place phenomenon, we conducted hierarchical regression analyses. Age, gender, depressive symptoms (PHQ-9), lifetime suicidal ideation/behavior (SBQ-R Item 1), 12-month suicidal ideation (SBQ-R Item 2), anxiety sensitivity (ASI), and obsessive-compulsive symptoms (OCI-R) served as independent variables. HPPI scores were used as the dependent variable. We calculated the analyses separately for the Online Sample and the Mobility Impaired Sample. There was no violation of the multicollinearity assumption as all values of tolerance were > 25, and all variance inflation factor values were <5. Assuming a medium-sized effect (*f* ^2^ = 0.15), an alpha error level of 5%, 7 predictors and sample sizes of *N* = 257/*N* = 233, the test power was 1-ß≥ 0.80 and therefore sufficient according to Cohen (24). With the aim to additionally identify predictors of the presence of the HPP (no=0, yes=1), two multiple logistic regression analyses were calculated using the same predictor variables as in the hierarchical regression analyses described above. Derived odds rations (including 95% confidence interval, CI) are presented for each predictor variable in both models. We calculated the analyses separately for the Online Sample and the Mobility Impaired Sample.

## Results

### Descriptive statistics, correlations and group differences

The high place phenomenon was known to *n* = 161 (62.6%) participants (HPPI-score ≥1) in the Online Sample and to *n* = 91 (39.1%) participants in the Mobility Impaired Sample. More precisely, the high place phenomenon was known to *n* = 67 (48.2%) of lifetime non-ideators and to *n* = 95 (78.5%) of lifetime suicidal ideators in the Online Sample. In the Mobility Impaired Sample *n* = 34 (26.4%) of lifetime non-ideators and *n* = 57 (54.8%) of lifetime suicide ideators reported experiences with the high place phenomenon.

Descriptive statistics for each measure and correlations are presented in **Table 1**. Correlation analyses indicated that experiences with the high place phenomenon were associated with depression, lifetime suicidal ideation/behavior, 12-month suicidal ideation, obsessive-compulsive symptoms and anxiety sensitivity in both samples.

**Table 1:** Means, standard deviations and correlations of study variables.

			HPPI	
	Sample 1	Sample 2	Sample 1	Sample 2
	*M* (*SD*)		*r*	
PHQ-9	20.08 (6.69)	19.99 (7.38)	.431**	.393**
ASI	25.91 (12.01)	22.59 (15.52)	.148*	.223**
OCI-R	40.58 (11.74)	38.11 (12.53)	.316**	.291**
SBQ-R-Item 1	1.83 (1.30)	2.02 (1.53)	.320**	.453**
SBQ-R-Item 2	1.51 (0.94)	1.60 (1.09)	.412**	.534**
HPPI	2.56 (2.88)	1.58 (2.64)		

*M*, Mean, *SD*, Standard Deviation; *r*, Pearson correlation; ASI, Anxiety Sensitivity Index; HPPI, High Place Phenomenon Index; OBQ-R, Obsessive-Compulsive Inventory-Revised; PHQ-9, Patient Health Questionnaire; SBQ-R, Suicide Behavior Questionnaire-Revised.

**p < .01, *p < .05.

### Prediction of experiences with the high place phenomenon

The results of the hierarchical regression analyses are shown in **Tables 2**, **3**. In both samples, the severity of experiences with the high place phenomenon were predicted by obsessive-compulsive symptoms, after controlling for age, gender, depression, suicidal ideation/behavior and anxiety sensitivity. Anxiety sensitivity was not associated with the high place phenomenon in both analyses. Of note, in the Online Sample the HPP was also associated with younger age, male gender and depression. However, no respective associations were found in the Mobility Impaired Sample.

**Table 2:** Prediction of HPP by depression, anxiety sensitivity, obsessive-compulsive symptoms and suicidal ideation/behavior in the online sample (*N* = 257).

Constructs	Model 1			Model 2		
	B	T	p	B	T	p
Age	-.069	-4.95	.001	-.071	-5.13	.001
Gender	.881	2.85	.005	.764	2.48	.014
PHQ-9	.095	3.60	.001	.067	2.26	.024
SBQ-R Item 1	.346	2.61	.010	.326	2.48	.013
SBQ-R Item 2	.492	2.52	.012	.504	2.61	.010
ASI	–	–	–	-.015	-1.04	.296
OCI-R	–	–	–	.045	2.84	.005
Model	Adj. R^2^ = .315 *F*(5, 251)=24.55 *p* < .001			Adj. R^2^ = .331 *F*(7, 249)=19.11 *p* < .001		
Change in R^2^	0.328 *p* < .001			0.021 *p=.*019		

ASI, Anxiety Sensitivity Index; HPPI, High Place Phenomenon Index; OBQ-R, Obsessive-Compulsive Inventory-Revised; PHQ-9, Patient Health Questionnaire; SBQ-R, Suicide Behavior Questionnaire-Revised.

**Table 3:** Prediction of HPP by depression, anxiety sensitivity, obsessive-compulsive symptoms and suicidal ideation/behavior in the mobility impaired sample (*N* = 233).

Constructs	Model 1			Model 2		
	B	T	p	B	T	p
Age	.015	1.05	.293	.013	0.92	.358
Gender	.360	1.31	.191	.398	1.45	.146
PHQ-9	.040	1.75	.081	.004	0.14	.887
SBQ-R Item 1	.352	3.13	.002	.390	3.48	.001
SBQ-R Item 2	.881	5.0^	.001	.852	4.86	.001
ASI	–	–	–	-.003	-0.26	.793
OCI-R	–	–	–	.036	2.28	.024
Model	Adj. R^2^ = .351 *F*(5, 226)=25.94 *p* < .001			Adj. R^2^ = .363 *F*(7, 224)=19.77 *p* < .001		
Change in R^2^	0.365 *p* < .001			0.017 *p=.*046		

ASI, Anxiety Sensitivity Index; HPPI, High Place Phenomenon Index; OBQ-R, Obsessive-Compulsive Inventory-Revised; PHQ-9, Patient Health Questionnaire; SBQ-R, Suicide Behavior Questionnaire-Revised.

The results of the multiple logistic regression analyses are shown in **Table 4**. In the Online Sample, the presence of the high place phenomenon was predicted by younger age, male gender, depression and obsessive-compulsive symptoms. In the Mobility Impaired Sample only lifetime suicidal ideation/behavior was associated with the presence of the HPP.

**Table 4:** Results from a multiple logistic regression analyses predicting presence of HPP.

Construct	Online Sample			Mobility Impaired Sample		
	OR	(95% CI)	*p*	OR	(95% CI)	*p*
Age	0.93	0.90-0.96	.001	1.08	0.97-1.04	.602
Gender	2.51	1.34-4.72	.004	1.72	0.94-3.16	.077
PHQ-9	1.07	1.01-1.14	.017	1.05	0.99-1.11	.065
SBQ-R Item 1	1.28	0.96-1.70	.086	1.39	1.09-1.76	.007
SBQ-R Item 2	1.19	0.73-1.95	.476	1.32	0.88-1.95	.155
ASI	0.99	0.97-1.03	.968	0.99	0.97-1.02	.867
OCI-R	1.04	1.00-1.07	.045	1.01	0.97-1.04	.648

ASI, Anxiety Sensitivity Index; HPPI, High Place Phenomenon Index; OBQ-R, Obsessive-Compulsive Inventory-Revised; PHQ-9, Patient Health Questionnaire; SBQ-R, Suicide Behavior Questionnaire-Revised.

## Discussion

In the present study, experiences with the high place phenomenon (HPP) were examined in two samples of Iranian adults. The HPP was known to 39% to 62% of all participants. Almost identical prevalence rates have been found in studies from Germany and the USA (1–3), which indicates that the HPP might be a ubiquitous phenomenon that is not restricted to Western societies. Therefore, the current findings complement other studies that have already demonstrated the cross-cultural spread of further forms of intrusive thoughts and impulses (9, 25). Consistent with previous research, this study also found that individuals with and without a history of suicidal ideation or behavior reported experiencing HPP.

In line with expectations, the severity of the HPP was associated with obsessive-compulsive symptoms in both samples, even after controlling for depression, suicidal ideation/behavior and anxiety sensitivity. However, the presence of the HPP was only associated with obsessive-compulsive symptoms in the Online Sample, but not in the Mobility Impaired Sample. Nonetheless, the result pattern supports the conceptualization of the HPP as being part of the phenomenological field of (subsyndromal) OCD symptoms. Of note, anxiety sensitivity was not associated with the HPP in either of the two samples investigated in the current study (cf. 1, 2). This calls into question whether the HPP is to be understood as a misinterpreted safety signal – as postulated by Hames et al. (3) – or whether the HPP eludes a clear functionality and is rather to be understood as a randomly occurring unwanted intrusive thought. In this sense, it must also be emphasized that the HPP is not per se a suicidal obsession. In principle, intrusions are not psychopathological; rather, normal intrusions can develop into distressing obsessions if they are interpreted as significant and threatening (6).

A few other findings of the current study seem noteworthy. First, experiences with the HPP were less often reported in the Mobility Impaired subsample (39%) compared to the current Online Sample (62%), as well as a German online sample (59.8%; 1). One reason for this could be that individuals with mobility impairment are less often triggered by environmental cues of height, making the experience less common for them. In a similar vein, lower prevalence rates have been found in previous studies focusing on individuals suffering from height and flight phobia (43%-45%; 1, 2), who possibly show some kind of avoidance behavior regarding heights. However, at the given moment this interpretation is pure speculation. Second, associations between the HPP and younger age and male gender have not been found in previous studies on the phenomenon. It is unclear how this finding came about, and we would caution against far-reaching interpretations. In principle, future studies should investigate the association between exposure to heights and the occurrence of the phenomenon – as this could also explain the link with age and gender. Third, the results of the hierarchical regression analyses and the multiple logistic regression analyses differ, particularly in the mobility-impaired sample. It may be that different factors are associated with the severity and occurrence of HPP, but at this point in time, it cannot be ruled out that methodological aspects (including sample size) are responsible for the diverging result patterns. Replication studies on larger samples are needed before further conclusions can be drawn.

### Clinical implications

Overall, it is important to understand and conceptualize the HPP differently depending on whether suicidal ideation is present or absent. In the context of a suicidal crisis, the HPP is likely to be experienced as ego-syntonic and less distressing, whereas the HPP outside of a suicidal crisis is more likely to be experienced as ego-dystonic and distressing [cf. (7, 26)]. In clinical practice, great care must therefore be taken to explore the context in which the HPP occurs and how it is experienced by the individual. In the vast majority of cases, the HPP can be normalized as an everyday phenomenon, while in some cases it will be understood as an expression of a suicidal obsession or as an expression of a suicidal crisis. Depending on the case conceptualization, a very different therapeutic approach results: If the HPP is understood as a common experience or a suicidal obsession, suicide-focused interventions may not be appropriate. Interventions such as safety planning or means restriction counseling [cf. (27)] could even have unintended effects. They might turn into compulsive reassurance-seeking rituals, which could reinforce and maintain the problem. In such cases, exposure therapy techniques (6) should be used instead. These methods help individuals learn that HPP-related thoughts and impulses are not dangerous and do not increase the risk of corresponding actions. On the other hand, exposure-based interventions are not appropriate when individuals experience the HPP in the context of a suicidal crisis. In these cases, strategies that reduce contact with suicidal cues must be chosen [such as means restriction techniques; (28)].

### Limitations

The results of the current study should be interpreted with consideration of the following limitations: First, the use of two self-selected *ad hoc* samples comprising highly educated Iranian adults limits the generalizability of the results. As such, the findings may not extend to less-educated or rural populations in Iran, nor necessarily to other non-Western cultures. Especially since a self-selection bias cannot be ruled out. In future studies either a representative population sample or a probabilistic sampling method should be used. Second, the cross-sectional design of the study limits any conclusions about causality or temporal ordering of effects. It is therefore unclear whether having obsessive intrusive thoughts leads to more HPP experiences, or if frequently experiencing HPP might in some way sensitize people to OCD-like thoughts – or whether a third factor contributes to both. Longitudinal studies are necessary to understand the temporal relationship between the two phenomena. Third, since all measures were self-report questionnaires taken in the same survey session there is a possibility that some of the associations found within the current study are inflated [cf. common-method bias; (29)]. A replication study is needed in which the different constructs are assessed at (short) intervals using both self-report measures as well as interview measures. Fourth, future studies of individuals with mobility impairments should collect more descriptive information and examine more closely the extent to which exposure to heights occurs or has occurred despite the impairments. Such data would not only enable a more accurate characterization of the sample and internal comparisons but could also provide information on whether HPP is related to actual altitude exposure.

## Conclusions

In sum, there is accumulating evidence that the high place phenomenon is a common, cross-cultural experience that is known to both individuals with lifetime suicidal ideation as well as individuals without lifetime suicidal ideation. The association between obsessive-compulsive symptoms and the HPP speaks to a conceptualization of the HPP as being part of the phenomenological field of (subsyndromal) OCD symptoms.

## Data Availability

The raw data supporting the conclusions of this article will be made available by the authors, without undue reservation.
